# Stability difference of each chromosome in Chinese Hamster Ovary cell line

**DOI:** 10.1186/1753-6561-9-S9-P1

**Published:** 2015-12-14

**Authors:** Noriko Yamano, Toshitaka Kumamoto, Mai Takahashi, Jana Frank, Masayoshi Onitsuka, Takeshi Omasa

**Affiliations:** 1 Institute of Technology and Science, Tokushima University, Tokushima, 770-8506, Japan; 2 Graduate School of Advanced Technology and Science, Tokushima University, Tokushima, 770-8506, Japan; 3 Graduate School of Engineering, Osaka University, Osaka, 565-0871, Japan

## Background

The use of biopharmaceutical products that comprise therapeutic antibodies are increasing in the pharmaceutical industry. Chinese hamster ovary (CHO) cell lines are widely used in the field of pharmaceutical industry to produce therapeutic antibodies. CHO DG44 cell line is a dihydrofolate reductase (DHFR)-deficient line, which is frequently used as a host cell while applying the gene amplification method. Since the chromosomes of CHO cells are unstable, variation in chromosome number occurs in these cells. This study focused on the dynamics of each chromosome during changes in the number of chromosomes. Clones from the CHO genomic bacterial artificial chromosome (BAC) library, a powerful tool that is expected to cover the entire CHO DG44 genome [1], were used to distinguish the location of individual chromosomes. In addition to the BAC clones previously known to correspond to the chromosomal locations of CHO DG44 and CHO K1 cell lines [2], sequences of 304 BAC clones that can identify the chromosomal locations in CHO DG44 cell line have been determined in this study. Therefore, our study leads to an understanding of the DNA sequence of individual chromosomes in the genome and the stability of each chromosome to establish high-producing CHO cell line.

## Materials and Methods

CHO DG44 (CHO-DR1000L-4N) cell line was cultured as described previously [3]. Six cell lines were isolated fromCHO DG44: three with 20 (normal) and three with over 30chromosomes. BAC-fluorescence in situ hybridization (FISH) technique was carried out as described previously [1-4] to identify each chromosome and analyze the rearrangement of chromosomes in these cell lines. HiSeq sequencing system was used by Takara Bio Inc. (Shiga, Japan)to analyze the BAC clone insert DNA sequences. Host cell (Escherichia coli str. K12 substr. DH10B)-derived sequences and the vector (pBAC-Lac)-derived sequences were omitted from the results. Edena v3 software was used to assemble the sequencing results.

## Results and Discussion

While most CHO DG44 cells showed a chromosome number of around 20 (named A to T in order of decreasing length [1]), 4% of the cells had more than 30 chromosomes. A comparison between the two groups revealed that the copy number of specific chromosomes increased or decreased with an overall change in the chromosome number (Table 1). In particular, the copy number of chromosome D was found to regularly decrease despite an increase in the total chromosomes in a cell. On the other hand, chromosomes A or B, E, F, G, H, I, K, M, O, Q, and S doubled in cell lines with a high chromosome number. Chromosomes of the CHO cell lines are known to rearrange significantly from the original Chinese hamster cells, with the exception of chromosomes A and B [2]. Chromosomes A and B are the only homologous chromosome pair found in CHO cells [2]. These are considered to be chromosome 1 and stable in CHO cells.

**Table 1:** Copy number of individual chromosomes in CHO cells with chromosome number variation.

Chromosome ID	Chromosome number in a cell	
	**20**	**over 30**
**A, B**	**2 (100%)**	**3 (100%)**
**C**	**1 (100%)**	**1 (80100%), 2 (20%)**
**D**	**0,(55%), 1 (45%)**	**0 (100%)**
**E**	**1 (100%)**	**2 (100%)**
**F**	**1 (100%)**	**2 (100%)**
**G**	**1 (100%)**	**2 (100%)**
**H**	**1 (100%)**	**2 (100%)**
**I**	**1 (100%)**	**1 (10%), 2 (80%), 3 (10%)**
**J**	**2 (100%)**	**2 (100%)**
**K**	**1 (100%)**	**1 (40%), 2(60%)**
**L**	**1 (83%), 2 (17%)**	**1 (71%), 2 (29%)**
**M**	**1 (100%)**	**2 (100%)**
**N**	**0 (100%)**	**0 (80%), 1 (20%)**
**O**	**1 (100%)**	**9 (95%)**
**P**	**1 (60%)**	**1 (40%)**
**Q**	**1 (100%)**	**2 (100%)**
**R**	**2 (100%)**	**2 (100%)**
**S**	**1 (100%)**	**1 (17%), 2 (83%)**
**T**	**0 (45%), 1(55%)**	**1(100%)**

Chromosomes are listed in order of decreasing length (from A to T). Orange, blue, and pink cells in the chromosome ID column represent an increase, decrease, and no change in number of the corresponding chromosome respectively.

**Figure 1:**
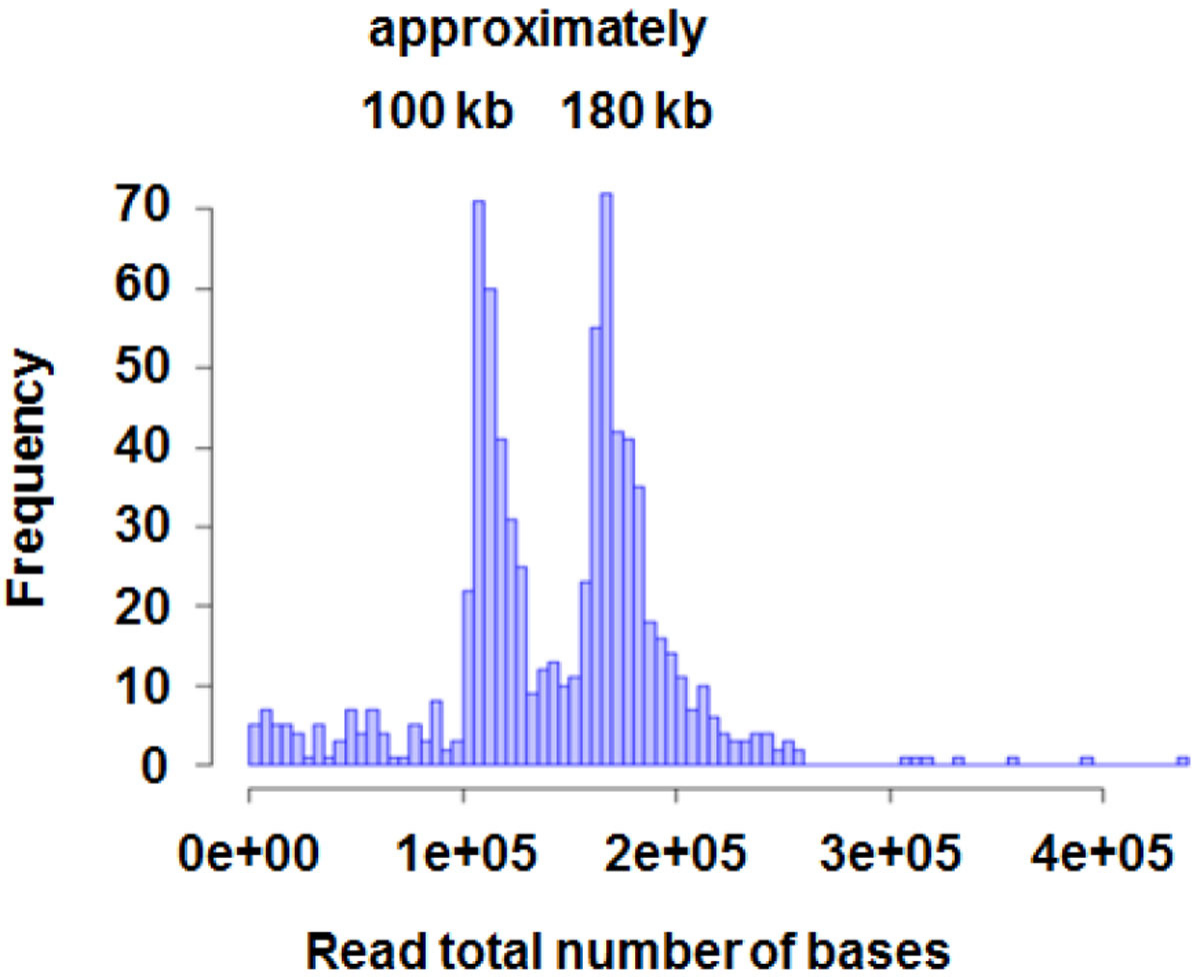
**Total number of DNA bases in each BAC clone insert determined by HiSeq sequencing**.

Further analyses were performed to determine the sequences of the 304 BAC clone inserts. The size distribution of BAC DNA inserts estimated by sequence assembling results is presented in the figure (Figure 1). By determining the sequence of BAC clone DNA, genome sequence of each chromosome in CHO DG44 and CHO K1 cell lines was revealed. As a next step, it is important to construct a screening system for isolating specific BAC clones containing particular sequences to find gene locations in the individual chromosomes.

## Conclusions

Our results suggest that chromosome stability differs across individual chromosomes in CHO cells. In recent years, site-specific integration of expression vectors by gene targeting has become a new approach to construct stable cell lines. Overlapping sequences on stable chromosomes appear to be attractive candidate locations for establishing high-yielding CHO cell lines.

Acknowledgements

This work was partly funded by a grant for the Project focused on developing key technology of discovering and manufacturing drug for next-generation treatment and diagnosis from the Ministry of Economy, Trade and Industry of Japan and partly by a Grant-in-Aid for Scientific Research from the Japan Society for the Promotion of Science (JSPS) (No.26630433, 26249125).
